# Evaluation of a commercial ELISA kit for detection of antibodies against *Toxoplasma gondii* in serum, plasma and meat juice from experimentally and naturally infected sheep

**DOI:** 10.1186/1756-3305-6-85

**Published:** 2013-04-05

**Authors:** Sabine B Glor, Renate Edelhofer, Felix Grimm, Peter Deplazes, Walter Basso

**Affiliations:** 1 Institute of Parasitology, Vetsuisse Faculty, University of Zurich, Winterthurerstrasse 266a, Zurich, CH-8057, Switzerland; 2 Department of Pathobiology, Institute of Parasitology and Zoology, University of Veterinary Medicine, Veterinärplatz 1, Vienna, 1210, Austria

**Keywords:** *Toxoplasma gondii*, Sheep, Diagnosis, ELISA, IFAT, Indirect haemagglutination, Antibodies, Meat juice

## Abstract

**Background:**

Toxoplasmosis is one of the most common food borne zoonoses worldwide, and can be a serious life-threatening disease in the congenitally infected fetus and in immunosupressed patients. Among food animals, sheep along with goats and pigs possess the highest incidence of *T. gondii* cysts in meat, and play a major role as a source of human infection.

**Methods:**

In this study, a new commercial ELISA kit (PrioCHECK® Toxoplasma Ab SR, Prionics Schlieren-Zurich, Switzerland) for the detection of anti-*T. gondii* antibodies in serum, plasma and meat juice of sheep, was evaluated by comparing it with the indirect fluorescent antibody test (IFAT), indirect haemagglutination test (IHA) and real-time PCR, on samples from experimentally inoculated and naturally exposed sheep.

**Results:**

The commercial ELISA detected the infection status in 50% and 100% of sheep orally inoculated with 10,000 *T. gondii* oocysts (n = 6), from two or three weeks post infection (wpi), respectively, both on serum and plasma samples. Meat juice from all experimentally inoculated sheep collected at slaughter (12 wpi) showed positive ELISA values. In naturally exposed sheep (n = 396), the ELISA showed a very good agreement with IFAT (*kappa* = 0.91-1.0) and IHA (*kappa* = 0.96-1.0) performed on serum; and a positive correlation was observed between ELISA values and IFAT titers. By a Receiver Operating Characteristics (ROC) curve analysis, the commercial ELISA had relative sensitivities between 93.33% and 100%, and relative specificities between 96.87% and 100% respect to IFAT and IHA, depending on the considered cut-off value and animal groups tested. Furthermore, the ELISA correctly recognized all animals reacting positive in real-time PCR. The ELISA results on meat juice agreed with those on serum samples in all experimentally inoculated animals, and in 94 out of 96 (97.9%) naturally exposed sheep, when meat juice was tested at a 1:10 dilution.

**Conclusion:**

The commercial ELISA kit evaluated in this study could represent a valuable tool to improve the surveillance and reporting system for *T. gondii* in sheep populations at the farm level or for diagnosis at the slaughterhouse, contributing to the control of this widespread zoonosis.

## Background

*Toxoplasma gondii* is a worldwide-distributed, cyst-forming protozoan parasite that affects warm-blooded animals and humans. Felids, the only known definitive hosts, shed oocysts in their faeces. These oocysts sporulate in the environment and represent the main source of infection for grazing animals. In sheep, infection with *T. gondii* is considered an important cause of abortion and stillbirth but subclinical infections are also very common. Worldwide, seroprevalences ranging from 4% to 95% have been reported for sheep [1,2]. In these animals, the parasite can persist asymptomatically in the form of bradyzoite-containing tissue cysts, mainly in the brain and muscles [3]. Among food animals, sheep along with goats and pigs, possess the highest incidence of cysts in meat, and play an important role as a source of infection for humans [2,4,5]. According to a multicentre case–control study among pregnant women in Europe, consumption of inadequately cooked or cured meat was the risk factor that most strongly predicted acute infection with *T. gondii*. Between 30% and 63% of infections in different centres were attributed to consumption of meat products and only 6% to 17% to soil contact [6]. Although infection with *T. gondii* in humans is frequently asymptomatic, it can be life-threatening for congenitally-infected as well as for immunosuppressed patients [7]. Therefore, the implementation of new meat safety strategies is an important issue for prevention of *T. gondii* transmission to humans [8]. In recent years, in order to improve data collection and to better understand the significance of toxoplasmosis, a scientific panel appointed by the European Food Safety Authority (EFSA) recommended that monitoring of the pre-harvest sector in sheep, goats, pigs and game should occur. They pointed out the need for reference materials and reagents and for well characterized diagnostic methods to be applied to food and animals [9].

So far, diagnostic tools available to detect *T. gondii* infection in sheep include direct methods such as histopathology, immunhistochemistry, polymerase chain reaction (PCR) and bioassays, and indirect (serological) methods, based on the detection of antibodies against the parasite. Serological tests (e.g. indirect fluorescent antibody test (IFAT), enzyme-linked immune sorbent assay (ELISA), modified agglutination test (MAT), western blot (WB), latex agglutination test (LAT) and indirect hemagglutination test (IHA) are generally highly sensitive and have been largely used worldwide. Recently, the performance of the different serological tests available for detecting antibodies against *T. gondii* in sheep was compared and discussed [1].

In an effort to facilitate implementation of new, simple reliable tests we evaluated a new commercial ELISA test kit (PrioCHECK® Toxoplasma Ab SR, Prionics Schlieren-Zurich, Switzerland) for detection of anti-*T. gondii* antibodies in serum and meat juice of sheep, by comparing it with the indirect fluorescent antibody test (IFAT), indirect haemagglutination test (IHA) and real-time PCR, using samples derived from naturally exposed and from experimentally infected sheep.

## Methods

### Experimental infection of sheep with *T. gondii*

#### Parasites

Experimental infections were performed with the CZ Tiger *T. gondii* isolate (type II) originally obtained from the faeces of a captive Siberian tiger (*Panthera tigris altaica*) kept at the Dvůr Králové Zoo in the Czech Republic in 2005 by Dr. B. Koudela, and since then maintained by passages between mice and cats [10].

#### Sheep (Group 1)

To obtain reference material (serum, plasma, meat juice, brain and muscle tissue), nine 5–7 month-old *T. gondii* seronegative sheep were purchased from a sheep breeder in Kemptthal, Zurich, Switzerland. Six animals were orally inoculated with a suspension containing 10,000 *T. gondii* oocysts of the CZ-Tiger isolate in phosphate buffered saline (PBS). The three remaining sheep received only PBS and served as negative, non-infected controls. All nine animals were clinically monitored over 12 weeks, and blood was taken from the *vena jugularis externa* at weeks 0 (inoculation day), 1, 2, 3, 4, 6, 8 and 12 (euthanasia) after inoculation in tubes with and without EDTA. Plasma and serum, respectively, were separated, fractioned, and stored at -20°C until use. All six inoculated sheep had fever (40.2 up to 41.8°C) for 6 or more days between days 3 and 11 post inoculation (dpi), and some of them showed serous nasal discharge, cough, tachypnoea and soft faeces from 4 to 6 dpi, and apathy from 4 to 13 dpi. The non-inoculated control animals displayed normal clinical parameters during the whole study. At euthanasia, samples from hind limb muscles were obtained, frozen at -20°C and thawed, and the generated meat juice was collected and conserved at -20°C for serology. Additionally, brain and hind limb muscles samples were collected for real-time PCR analysis.

All animal experiments were authorized by the Cantonal Veterinary Office of Zurich, Switzerland (permission no. 106/2010 and 130/2012) and comply with the current laws of the country.

### Field samples from sheep

#### Swiss sheep (Group 2)

Samples of serum, striated muscle (*Musculus masseter*) and brain were obtained from 96 sheep of different breeds and age at the slaughterhouse of the Animal Hospital of the Vetsuisse Faculty, Zurich, Switzerland. Meat juice was obtained from *M. masseter* for serological analysis as described above, and muscle and brain samples were stored at -20°C for real-time PCR analysis.

#### Recent natural infections

In an attempt to select *Toxoplasma*-free sheep for an experiment other than the one described here, we bought nine animals from two Swiss farms (seven and two animals from farms 1 and 2, respectively), which initially tested negative for *T. gondii* infection by PrioCHECK® Toxoplasma Ab SR ELISA (day 0). These animals were then kept indoors and retested by ELISA, IFAT and IHA 25 and 35 days later. However, the seven sheep derived from farm 1 seroconverted during this quarantine. They were, therefore, slaughtered at day 35 and their samples were included in Group 2.

#### Austrian sheep (Groups 3 and 4)

Serum samples from 300 sheep aged 0.5 to 10 years, from different breeds and origin, were randomly obtained from a larger sampling of 4,079 animals performed in Austria, where antibodies against *T. gondii*, were detected in 66% of sheep [5]. There were 150 samples derived from sheep with positive (IFAT titer ≥1:40) *T. gondii* antibody titers (IFAT titer: 1:40 [n = 10], 1:80 [n = 16], 1:160 [n = 22], 1:320 [n = 30], 1:640 [n = 19], 1:1,280 [n = 30], 1:2,560 [n = 15] and ≥1:5,120 [n = 8]) (Group 3), and 150 samples from seronegative animals (IFAT titer <1:40) (Group 4).

### Serological analysis

#### Enzyme-linked immunosorbent assay (ELISA)

A commercial ELISA kit (PrioCHECK® Toxoplasma Ab SR, Prionics, Schlieren-Zurich, Switzerland) for detection of IgG antibodies against *T. gondii* in small ruminants was evaluated. The kit includes ELISA plates coated with cell culture derived *T. gondii* tachyzoite-antigen, a peroxidase-labelled anti-small ruminant secondary antibody, tetramethyl benzidine (TMB) as a chromogenic substrate, control sera and buffer solutions.

Serum (Groups 1–4) and plasma (Group 1) samples were tested at a 1:100 dilution in sample diluent buffer, and meat juice samples (Groups 1 and 2) at 1:10 and 1:20 dilutions. Optical density (OD) was measured at 450 nm (reference filter 620 nm) and the test results were interpreted by calculating, for each sample, a percentage of positivity (PP) value relative to the OD of the positive control (PP Sample = OD450 nm Sample / OD450 nm Positive Control x 100). A PP value ≥ 20 was *a priori* regarded as positive (as suggested by the manufacturer), and re-evaluated after Receiver Operating Characteristic (ROC) curve analysis; PP values below 20 were considered negative.

#### Indirect fluorescent antibody test (IFAT)

For antigen, tachyzoites of *T. gondii* RH strain were cultivated in human foreskin fibroblasts (HFF) in Dulbecco’s Modified Eagle Medium supplemented with 10% foetal calf serum, 1% L-glutamine and 1% penicillin-streptomycin-fungizone (Sigma-Aldrich Co., Switzerland) at 37°C and 5% CO_2_. After harvest and purification, *T. gondii* tachyzoites were spotted onto IFAT slides and fixed in cold acetone. Serum and meat juice samples (Groups 1 and 2) were diluted in PBS and tested at two-fold dilutions from 1:40 up to 1:5,120 (serum) or 1:80 (meat juice). Fluorescein isothiocyanate (FITC) rabbit anti-sheep IgG (whole molecule) conjugate (Sigma-Aldrich, Steinheim, Germany) was used as a secondary antibody at a 1:100 dilution in PBS. Only a complete peripheral fluorescence of the tachyzoites was interpreted as positive, while polar or lack of fluorescence was judged as negative. Samples showing no fluorescence at a dilution of 1:40 were judged as negative and not tested at a lower dilution. Known positive and negative control sera were included in each reaction series.

Serum samples from Groups 3 and 4 were analyzed previously by IFAT at the Institute of Parasitology of the Veterinary Medicine University in Vienna, Austria, essentially as described above, but using FITC conjugate donkey anti-sheep IgG (heavy and light chains) (Jackson Immuno Research Inc., West Grove, PA, USA) at a dilution of 1:150 as secondary antibody.

#### Indirect haemagglutination test (IHA)

A commercial indirect haemagglutination test kit (ELI.H.A Toxo, ELITech Group, Switzerland) was used to detect anti-*T. gondii* IgG in sheep sera from Groups 1–4 (Group 1 was tested at 1, 2, 3 and 12 wpi.), at a dilution of 1:80 (recommended cut-off by the manufacturer). To avoid false positive reactions, an adsorption of the natural anti-sheep agglutinins, and a treatment with 2-mercaptoethanol (2-ME) to inactivate IgM, were performed for all tested sera. A cloudy red/brown deposit, coating the well, representing haemagglutination, was judged as a positive reaction and a ring at the bottom of the well was judged as a negative reaction (no haemagglutination).

### Real-time PCR

#### Sample preparation – digestion and DNA-extraction

DNA was extracted from brain and muscle (hind limb muscles in Group 1, *M. masseter* in Group 2) with the DNeasy Blood and Tissue Kit (QIAamp DNA Mini Kit, Qiagen GmbH, Hilden, Germany). First, one gram of tissue was digested with 1.8 ml ATL buffer and 200 μl proteinase K (20 mg/ml) (Roche Diagnostics GmbH, Mannheim, Germany) overnight on a shaker at 56°C [11]. Subsequently, 200 μl of digested tissue were further processed with the standard protocol, according to the manufacturer’s instructions. The DNA extract was frozen at -20°C until used for real-time PCR.

#### Real-time PCR analysis

Real-time PCR analysis, targeting the 529-bp repeat element (RE) of *T. gondii*, was performed, using primers described by Cassaing et al. (2006) [12] with slight modifications: Primer TgF1 : 5‘-AAGGCGAGGGTGAGGATGA-3’, primer TgR1 : 5‘-TCGTCTCGTCTGGATCGCAT-3’ and a new designed TaqMan probe TgS1: FAM 5’-TTCCGGCTTGGCTGCTTTTCCTG3’-BHQ1, in a final reaction volume of 25 μl, including 5 μl of template DNA, 0.5 μl of each primer, 0.5 μl probe, 12.5 μl TaqMan® Universal PCR Master Mix (Applied Biosystems, Roche, Branchburg, New Jersey, USA) and 6 μl 10 mM TrisHCl pH 7.5 per reaction. Every sample was tested in duplicate. PCR inhibition was ruled out by a parallel plasmid specific PCR in spiked samples (internal control). All plates included a positive control (DNA from cell culture derived *T. gondii* tachyzoites), ddH_2_O as a negative control and a standard curve for *T. gondii* DNA quantification. The detection limit is approximately 0.1 parasites per reaction [12]. Internal control for specificity at our lab revealed no amplification when DNA samples from related protozoa such as *Sarcocystis miescheriana, S. cruzi, S. hirsuta*, *Hammondia heydorni, Hammondia hammondi, Neospora caninum* and *Besnoitia besnoiti* were tested. Cycling was started with 2 min incubation at 50°C and 10 min at 95°C, followed by 40 cycles of 95°C for 15 sec and 60°C for 60 sec. Amplification was carried out on an Applied Biosystems 7900HT Fast Real-Time PCR System instrument. Data were analysed using the TaqMan®-Software Sequence Detection Systems, Version 2.3 (SDS 2.3), Applied Biosystems. An assay was considered positive for *T. gondii* if at least one of the duplicate tests was positive (threshold value C_T_ <40).

### Statistical evaluation

Statistical analyses were performed using MedCalc for Windows, version 12.3.0.0. (MedCalc Software, Mariakerke, Belgium). To evaluate the agreement among different tests, inter-rater agreement (kappa) was calculated and kappa values (κ) were considered as follows: poor agreement (ĸ < 0.20); fair agreement (ĸ = 0.21–0.40); moderate agreement (ĸ = 0.41–0.60); good agreement (ĸ = 0.61–0.80); or very good agreement (ĸ = 0.81-1.00) (MedCalc Software, Mariakerke, Belgium). To evaluate the ability of the commercial ELISA to discriminate between infected and non-infected animals, and to compare its diagnostic performance with that of other serological and molecular tests, Receiver Operating Characteristics (ROC) curve analysis was used and relative sensitivity and specificity of the ELISA were determined, considering a 95% confidence interval [13]. According to an arbitrary guideline, the area under the curve (AUC) was considered: non-informative (AUC = 0.5); low accurate (0.5 < AUC ≤ 0.7); moderately accurate (0.7 < AUC ≤ 0.9); highly accurate (0.9 < AUC < 1) or perfect (AUC = 1) [14].

## Results

### Experimentally infected sheep (Group 1)

**Figure 1:**
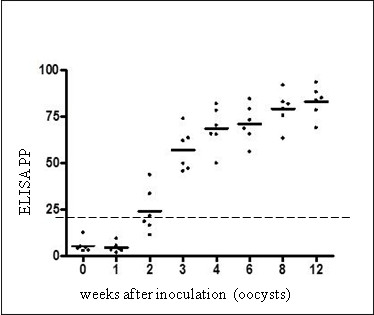
**Time course of specific IgG in sera of sheep experimentally inoculated with *T. gondii *oocysts (Group 1), tested by PrioCHECK® Toxoplasma Ab SR ELISA at different points post inoculation.** PP: Index for percentage of positivity; dashed line: cut-off PP 20.

**Figure 2:**
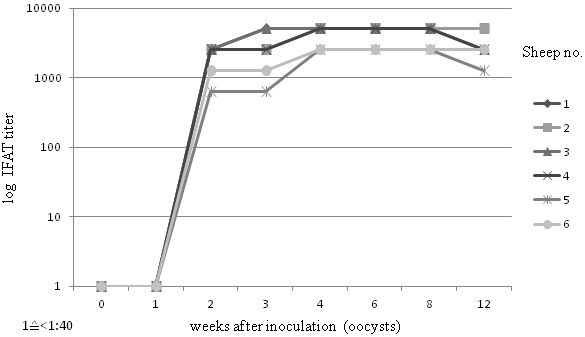
**Time course of specific IgG in sera of sheep experimentally inoculated with *T. gondii *oocysts (Group 1), tested by IFAT at different points post inoculation.**

Using the new PrioCHECK® Toxoplasma Ab SR ELISA kit, positive values (PP ≥20) in serum were first detected at 2 weeks post inoculation (wpi) in three sheep. From 3 wpi, all six experimentally infected animals showed positive values until the end of the study (Figure 1). All six sheep showed positive reaction by both IFAT and IHA from 2 wpi; IFAT antibody titers rose from 1:640 to 1:2,560 at 2 weeks p.i., to 1:1,280 to ≥ 1:5,120 at slaughter (Figure 2). The infection status in this group was also supported by the occurrence of clinical signs in all six sheep and by positive real-time PCR results on brain and/or muscle tissue in five out of six animals. No significant differences were observed in the longitudinal study between ELISA values in serum and plasma samples.

Meat juice samples obtained from all six sheep showed PP ELISA values ≥20 both at dilutions 1:10 (PP 65.9-92.9) and 1:20 (PP 48.4-70.1) and IFAT titers ≥1:80. The non-infected control animals gave negative results by all tests: ELISA; IFAT; IHA; and real-time PCR.

### Field samples (Groups 2–4)

#### Sheep from Switzerland (Group 2)

In this group, 18 out of 96 sheep showed positive results in serum by the commercial ELISA. The same 18 animals reacted positive in IFAT (titers 1:160 [n = 1], 1:640 [n = 4], 1:1,280 [n = 6], 1:2,560 [n = 3] and ≥1:5,120 [n = 4]) and IHA. A positive correlation was found between ELISA PP values and IFAT titers (Additional file 1). Moreover, in 11 out of those 18 seropositive animals, a *T. gondii* infection was also confirmed by real-time PCR on brain (n = 6) and *M. masseter* (n = 7) tissues (Table 1). All remaining 78 seronegative sheep were negative in real-time PCR on both brain and muscle samples.

**Table 1:** **Real-time PCR for *T. gondii *in muscle and brain tissue samples (tested in duplicate [a, b]) from 18 seropositive (IFAT, IHA, PrioCHECK® Toxoplasma Ab SR ELISA) naturally exposed sheep (Group 2)**

**Sheep No.**	**Tissue**				**PCR result**	**ELISA PP value**	**IHA result**	**IFAT titer**
	**Brain**		**Skeletal muscle**					
	**PCR a (C**_**T **_**value)**	**PCR b (C**_**T **_**value)**	**PCR a (C**_**T **_**value)**	**PCR b (C**_**T **_**value)**				
01-1306	+ (34.76)	+ (36.30)	+ (32.16)	+ (32.80)	+	80.45	+	1:640
02-1308	-	-	-	-	-	36.17	+	1:160
03-1503	-	-	-	-	-	104.73	+	1:1280
04-1628	+ (32.80)	+ (33.96)	-	-	+	105.26	+	1:1280
05-1640	-	-	-	-	-	85.89	+	1:2560
06-1641	-	-	+ (29.38)	+ (29.16)	+	79.83	+	1:640
07-1646	+ (28.48)	+ (28.89)	-	-	+	73.91	+	1:1280
08-1649	-	-	-	-	-	38.26	+	1:1280
09-1651	-	-	-	-	-	85.46	+	1:1280
10-1653	+ (30.67)	+ (30.50)	-	-	+	95.74	+	1:2560
11-3879	-	-	+ (36.25)	+ (38.49)	+	85.32	+	≥1:5120
12-3880	-	-	+ (35.15)	+ (32.69)	+	91.87	+	≥1:5120
13-3883	-	-	-	-	-	74.31	+	1:2560
14-3885	-	-	+ (34.78)	+ (38.76)	+	47.41	+	1:640
15-3886	-	-	-	-	-	38.19	+	1:640
16-4947	-	+ (38.83)	-	-	+	101.33	+	≥1:5120
17-4948	-	-	+ (36.82)	+ (38.69)	+	79.08	+	≥1:5120
18-5785	+ (26.34)	+ (26.57)	+ (36.77)	+ (37.57)	+	26.32	+	1:1280
**Total positive**	**6/18**		**7/18**		**11/18**	**18/18**	**18/18**	**18/18**

Cut-offs considered: IFAT: 1:40; IHA: 1:80; ELISA PP 20. **C**_**T**_: Cycle threshold. (Real-time PCR results from seronegative sheep not displayed).

When ELISA was performed with meat juice, 20 or 18 out of 96 sheep showed a positive reaction at meat juice dilutions 1:10 or 1:20, respectively (Table 2). When comparing these results with those obtained with serum, it was observed that, when using meat juice at a 1:10 dilution, two additional animals reacted positively (i.e. two “false positives”). On the other hand, when using meat juice at a 1:20 dilution, 18 samples reacted positively: one negative animal using serum reacted now positive (i.e. one “false positive”) and one positive animal was now negative (i.e. one “false negative”). In both cases, we found that in 94 of 96 (97.9%) animals, the results obtained by testing meat juice agree with those obtained by testing serum. The same result was observed when meat juice-ELISA was compared with meat juice-IFAT (cut-off 1:40) (Table 2). Meat juice samples from all 18 animals reacting positively in serum-IFAT also showed positive titers in meat juice-IFAT (IFAT titers 1:40 [n = 3] and ≥1:80 [n = 15]).

**Table 2:** **Comparison of PrioCHECK® Toxoplasma Ab SR ELISA-results (cut-off PP 20) from 96 sheep from Switzerland (Group 2) to results obtained by IFAT, IHA (not performed with meat juice samples) and real-time PCR, and inter-rater agreement (kappa value)**

		**IFAT serum cut-off 1:40**			**IFAT serum cut-off 1:80**			**IHA serum cut-off 1:80**			**Real-time PCR brain/muscle**		
**A**		**positive**	**negative**	**total**	**positive**	**negative**	**total**	**positive**	**negative**	**total**	**positive**	**negative**	**total**
**ELISA serum**	positive	18	0	**18**	18	0	**18**	18	0	**18**	11	7	**18**
	negative	0	78	**78**	0	78	**78**	0	78	**78**	0	78	**78**
	total	**18**	**78**	**96**	**18**	**78**	**96**	**18**	**78**	**96**	**11**	**85**	**96**
	**Kappa**	1.0			1.0			1.0			0.719		
	**Standard Error**	0			0			0			0.0983		
	**95% CI**	1.0-1.0			1.0-1.0			1.0-1.0			0.526-0.911		
			**IFAT meat juice cut-off 1:40**			**IFAT meat juice cut-off 1:80**			**Real-time PCR brain/muscle**				
**B**			**positive**	**negative**	**total**	**positive**	**negative**	**total**	**positive**	**negative**	**total**		
**ELISA meat juice 1:10**	positive		18	2	**20**	15	5	**20**	11	9	**20**		
	negative		0	76	**76**	0	76	**76**	0	76	**76**		
	total		**18**	**78**	**96**	**15**	**81**	**96**	**11**	**85**	**96**		
	**Kappa**		0.934			0.826			0.659				
	**Standard Error**		0.0458			0.0746			0.102				
	**95% CI**		0.845-1			0.68-0.972		0.46-0.859					
**ELISA meat juice 1:20**	positive		17	1	**18**	15	3	**18**	11	7	**18**		
	negative		1	77	**78**	0	78	**78**	0	78	**78**		
	total		**18**	**78**	**96**	**15**	**81**	**96**	**11**	**85**	**96**		
	**Kappa**		0.932			0.890			0.719				
	**Standard Error**		0.0478			0.0619			0.0983				
	**95% CI**		0.838-1			0.769-1			0.526-0.911				

A: serum samples; B: meat juice samples at a dilution of 1:10 and 1:20. CI: confidence interval.

Comparative results by ELISA, IFAT, IHA and real-time PCR from the seven Swiss sheep in which a natural recent *T. gondii* infection was detected are displayed in Table 3. At day 0 we observed that all seven animals tested negatively by ELISA on serum but three animals already reacted positively in IFAT with low titers (1:40 [n = 2], 1:80 [n = 1]) and two of them were also positive in IHA. After 25 and 35 days, all animals were positive in the three serological tests and the infection could be additionally confirmed by real-time PCR (at day 35) in five out of seven animals.

**Table 3:** **Comparative results of seven sheep with recent natural *T. gondii *infection; serum tested by PrioCHECK® Toxoplasma Ab SR ELISA (cut-off PP 20), IFAT (cut-off 1:40) and IHA (cut-off 1:80) at day 0, 25 and 35 (slaughter) and real-time PCR of brain and skeletal muscle at day 35**

**Sheep no.**	**Time course (days)**	ELISA (PP)*	**IFAT (titer)**	**IHA**	**Real-time PCR brain**	**Real-time PCR skeletal muscle**
4947	0	- (3.69)	- (<1:40)	-		
	25	+ (89.02)	+ (≥1:5,120)	+		
	35	+ (101.33)	+ (≥1:5,120)	+	+	-
4948	0	- (3.28)	- (<1:40)	-		
	25	+ (82.43)	+ (≥1:5,120)	+		
	35	+ (79.08)	+ (≥1:5,120)	+	-	+
3879	0	- (4.24)	+ (1:40)	+		
	25	+ (78.86)	+ (≥1:5,120)	+		
	35	+ (85.32)	+ (≥1:5,120)	+	-	+
3880	0	- (6.20)	+ (1:80)	+		
	25	+ (86.23)	+ (≥1:5,120)	+		
	35	+ (91.87)	+ (≥1:5,120)	+	-	+
3883	0	- (9.07)	+ (1:40)	-		
	25	+ (38.32)	+ (≥1:5,120)	+		
	35	+ (74.31)	+ (1:2,560)	+	-	-
3885	0	- (5.88)	- (<1:40)	-		
	25	+ (44.29)	+ (1:1,280)	+		
	35	+ (47.41)	+ (1:640)	+	-	+
3886	0	- (12.39)	- (<1:40)	-		
	25	+ (31.39)	+ (1:1,280)	+		
	35	+ (38.19)	+ (1:640)	+	-	-

* PP: Index for percentage of positivity.

#### Sheep from Austria (Groups 3 and 4)

By the commercial ELISA, 139 out of 150 serum samples in Group 3 (IFAT positive) showed positive values (PP ≥ 20). Samples reacting negatively in ELISA had IFAT titers of 1:40 (n = 6), 1:80 (n = 4) and 1:320 (n = 1). A positive correlation was found between ELISA PP values and IFAT titers (Additional file 2). By IHA, 141 samples were recognized as positive (the nine negative samples in IHA had IFAT titers 1:40 [n = 5], 1:80 [n = 3], and 1:160 [n = 1]). Eight of the 11 samples that were negative by ELISA were also negative by IHA.

In Group 4 (IFAT negative), 148 out of 150 serum samples were also negative in ELISA, but two samples showed a positive reaction (PP = 21.05 and 26.12). All 150 samples were negative by IHA. Comparison of ELISA results with results obtained by IFAT and IHA are displayed in Table 4.

**Table 4:** **Comparison of PrioCHECK® Toxoplasma Ab SR ELISA-results (cut-off PP 20) from serum samples of 300 sheep from Austria (Groups 3 and 4) to results obtained by IFAT (cut-offs 1:40 and 1:80) and IHA and inter-rater agreement (kappa value)**

		**IFAT serum cut-off 1:40**			**IFAT serum cut-off 1:80**			**IHA serum cut-off 1:80**		
		**positive**	**negative**	**total**	**positive**	**negative**	**total**	**positive**	**negative**	**total**
**ELISA serum**	positive	139	2	**141**	135	6	**141**	138	3	**141**
	negative	11	148	**159**	5	154	**159**	3	156	**159**
	total	**150**	**150**	**300**	**140**	**160**	**300**	**141**	**159**	**300**
	**Kappa**	0.913			0.926			0.960		
	**Standard Error**	0.0235			0.0218			0.0162		
	**95% CI**	0.867-0.959			0.884-0.969			0.928-0.992		

### Statistical evaluation

The ELISA test kit was evaluated by comparing the obtained PP values of sheep samples to the results obtained by testing the same samples in IFAT, IHA and real-time PCR performing inter-rater agreement and ROC curve analysis. Considering Group 2 (sheep from Switzerland), the corresponding values for kappa were 1.0 when comparing serum-ELISA to serum-IFAT (cut-offs 1:40 and 1:80) and IHA, representing a very good agreement. Comparing serum-ELISA to real-time PCR, kappa value was 0.719, representing a good agreement. Calculated kappa values comparing ELISA performed on meat juice at a dilution of 1:10 and 1:20 to meat juice-IFAT (cut-off 1:40 and 1:80), were between 0.826 and 0.890 (at cut-off 1:80) and 0.934 and 0.932 (at cut-off 1:40), respectively, representing a very good agreement. The agreement observed between meat juice-ELISA and real-time PCR was good at both meat juice dilutions (κ =0.659 [1:10 dilution] and 0.719 [1:20 dilution]) (Table 2).

Considering results on sheep from Austria (Groups 3 and 4), when comparing the serum-ELISA with the other serological tests (IFAT cut-off 1:40 and 1:80 and IHA) on sera, the calculated kappa values were between 0.913 (IFAT cut-off 1:40) and 0.960 (IHA), representing a very good agreement of ELISA with both tests (Table 4).

The relative accuracies of the commercial ELISA, using serum and meat juice samples, calculated by ROC analysis are displayed in Table 5. Considering results obtained on serum samples from Group 2 (sheep from Switzerland), the commercial ELISA showed relative sensitivities and specificities of 100% with respect to both IFAT and IHA when a cut-off of 19.79 PP was selected, and a relative sensitivity of 100% and a relative specificity of 91.76% with respect to real-time PCR (All real-time PCR positive samples were recognized as positive by ELISA, but some samples with negative PCR results were serologically positive in ELISA, erroneously accounting for a lower specificity when this test is taken as the reference). Considering results on meat juice samples, the ELISA showed a relative sensitivity of 100% and a relative specificity of 98.72% when meat juice was diluted 1:10, IFAT (cut-off 1:40) was used as reference test, and the ELISA cut off was set at 20.29 PP.

**Table 5:** **ROC-analysis: Relative accuracies of PrioCHECK® Toxoplasma Ab SR ELISA (cut-off PP 20) in relation to IFAT, IHA and real-time PCR**

			**PrioCHECK® Toxoplasma Ab SR ELISA**		
**Reference test**	**Sample**	AUC/P*	**Selected cut-off in ELISA**	**% relative sensitivity (95% confidence interval)**	**% relative specificity (95% confidence interval)**
**A**					
IFAT (cut-off 1:40)	serum	1.000/<0.000	19.79	100 (81.5 - 100.0)	100 (95.4 – 100.0)
IFAT (cut-off 1:80)	serum	1.000/<0.000	19.79	100 (81.5 - 100.0)	100 (95.4 – 100.0)
IHA (cut-off 1:80)	serum	1.000/<0.000	19.79	100 (81.5 - 100.0)	100 (95.4 – 100.0)
Real-time PCR	brain/muscle	0.959/<0.0001	19.79	100 (71.5 - 100.0) ^§^	91.76 (83.8 - 96.6)^§^
IFAT (cut-off 1:40)	meat juice 1:10**	0.999/<0.0001	20.29	100 (81.5 - 100.0)	98.72 (93.1 - 100.0)
IFAT (cut-off 1:80)	meat juice 1:10**	0.996/<0.0001	27.64	100 (78.2 - 100.0)	97.53 (91.4 - 99.7)
IFAT (cut-off 1:40)	meat juice 1:20**	0.999/<0.0001	15.83	100 (81.5 - 100.0)	98.72 (93.1 - 100.0)
IFAT (cut-off 1:80)	meat juice 1:20**	0.998/<0.0001	27.45	100 (78.2 - 100.0)	98.77 (93.3 - 100.0)
**B**					
IFAT (cut-off 1:40)	serum	0.991/<0.0001	17.24	93.33 (88.1 - 96.8)	98.67 (95.3 - 99.8)
IFAT (cut-off 1:80)	serum	0.985/<0.0001	21.05	96.43 (91.9 - 98.8)	96.87 (92.9 - 99.0)
IHA (cut-off 1:80)	serum	0.998/<0.0001	21.05	97.87 (93.9 - 99.6)	98.74 (95.5 - 99.8)

A: Tests performed with samples from Swiss sheep (Group 2), B: Tests performed with samples from Austrian sheep (Groups 3 and 4).

*Significance level P (Area = 0.5); ** meat juice dilution; AUC: Area under curve.

^§^ Note: The apparent low specificity of ELISA when real-time PCR is taken as reference test might be related to the low diagnostic sensitivity of PCR techniques linked to the small size of the analyzed sample. All real-time PCR positive animals were recognized as positive by ELISA (100% relative sensitivity), but some serologically positive animals in ELISA had negative PCR results accounting for a (potentially erroneous) relative lower specificity of the ELISA.

When considering the serological results from sheep from Austria (Groups 3 and 4), the commercial ELISA showed a relative sensitivity between 93.33% and 96.43% and specificity between 96.87% and 98.67% with respect to IFAT (depending on the considered IFAT cut-off), and of 97.87% and 98.74%, respectively, with respect to IHA, when ELISA cut-offs between 17.24 and 21.05 PP were selected. ROC curve analysis revealed that ELISA on serum was highly accurate relative to IFAT cut-offs 1:40 and 1:80 (AUC = 0.991 and 0.985, respectively) and to IHA (AUC = 0.998). ROC curves and associated files can be provided from the authors upon request. In all performed analyses, the *p* value was < 0.0001 evidencing that this ELISA does have an ability to distinguish between infected and non-infected animals.

## Discussion

The evaluation of new diagnostic tools to determine *T. gondii* infection in animals is challenging because no “gold standard” reference methods (with 100% sensitivity and specificity) are available for mass scale samples. Some authors have suggested bioassay in cats or mice as the “gold standard” to confirm *T. gondii* infection [15-21], however, this method is expensive, time consuming and arguable with regard to animal welfare. Studies comparing the accuracy of multiple serological tests with bioassay-based reference standards have been reported in pigs [22], but not in other animal species such as small ruminants [1,23]. Recently, the evaluation of tests for *T. gondii* infection in intermediate hosts has been reviewed and some general recommendations about different statistical approaches (i.e. ROC analysis, titre-specific likelihood ratios and Bayesian latent class methods) have been made. In the present study, an ELISA test kit (PrioCHECK® Toxoplasma Ab SR) was evaluated on sheep of different breeds, age and origin with natural exposure to *T. gondii* and on experimentally inoculated sheep. Recently, an ELISA test kit based on the same technology was evaluated for its use in pigs, using a Bayesian latent class approach, and showed estimated sensitivity and specificity of 98.9% and 92.7%, respectively [24]. In this study, the infection status of the different groups of sheep was determined by testing plasma, serum, meat juice and tissues with different diagnostic reference tests (IFAT, IHA and/or real-time PCR) and these results were compared to those obtained using the new ELISA kit and analysed statistically. These reference tests have been widely used in epidemiological studies worldwide, alone or in combination [1,2], and they detect infection by different mechanisms: IFAT (a primary serological test) detects directly the antigen-antibody binding; IHA (a secondary serological test) detects the consequence of this binding: i.e. agglutination; and real-time PCR (a direct diagnostic method) detects the presence of parasite DNA in the tissues of infected animals. Using all three tests, in combination, increases diagnostic sensitivity. In our study, an almost perfect agreement was found when the commercial ELISA was compared to other serologic methods (IFAT and IHA) on serum samples. By ROC analysis, relative sensitivities and specificities of PrioCHECK® Toxo ELISA were 93.3-100%, and 96.9-100%, respectively, when an ELISA cut-off between PP = 17 and PP = 21 was considered. Therefore, the ELISA cut-off of PP = 20, suggested by the manufacturer, seems to be appropriate.

Sheep can develop very high levels of anti-*T. gondii* antibodies during acute infection with *T. gondii* and high IgG antibody titers can persist for months or years [21]. Testing experimentally infected sheep, we could detect ELISA values above the suggested cut-off (PP 20) from 2 to 3 wpi, persisting at high levels until the end of the experiment at 12 wpi. However, it appears that this test might fail to recognize infected animals at very early stages of infection, since at 2 wpi., only three out six animals reacted positive in ELISA, while all six animals were already positive in IFAT and IHA. Also, in the group of recently naturally infected sheep, we observed that seroconversion in some animals occurred later in ELISA than in IFAT or IHA. However, in a further experiment, nine sheep inoculated with 100,000 *T. gondii* oocysts (a dose ten times higher than that used in the present study), all nine animals showed antibody values above the cut-off from 2 wpi, suggesting that the infection dose could influence the diagnostic sensitivity of the test (unpublished results).

Several indirect methods have been proposed for the detection of anti-*T. gondii* antibodies in serum and plasma samples. In our study, we additionally tested meat juice as it could be a reasonable alternative to serum when testing carcasses and other samples for serological analysis are not available. Meat juice has been already used for the detection of antibodies against *T. gondii*[4,25,26]. Wingstrand *et al.* (1997) observed an excellent correlation between values obtained from meat juice and serum samples from pigs infected experimentally with *T. gondii,* when tested with an indirect in-house IgG ELISA using a lysate of *T. gondii* tachyzoites as antigen [25]. In the present study, we could also demonstrate a good correlation with sheep samples as, in 97.9% of the naturally exposed animals, the results obtained in ELISA by testing meat juice agreed with those obtained by testing serum. According to our results, a 1:10 dilution for meat juice (representing a 10 times lower dilution than that used for serum samples) appears to be adequate in order to guarantee a high sensitivity of the test. Although two additional animals showed a “false positive” reaction (compared to results on serum), all serum-positive animals were also positive using meat juice. Using a 1:20 meat juice dilution, only one animal showed a “false positive” reaction, but one “true positive” animal was not detected. When comparing ELISA results with IFAT results on meat juice, we observed significant differences according to the considered IFAT cut-off value (1:40 or 1:80). This could be due to low IFAT titers of the samples, often being no higher than 1:40 and, therefore, turning from a positive to a negative result when using a cut-off of 1:80. In order not to lose positive animals, we decided to set the cut-off for meat juice-IFAT at 1:40. Comparing this IFAT to ELISA (meat juice dilution 1:10), a relative sensitivity of 100% and relative specificity of 98.72% were obtained when the ELISA cut off was set at PP = 20. Therefore, we considered that the commercial ELISA can be also used on meat juice samples, and a meat juice dilution of 1:10 might be suggested for screening purposes.

In order to confirm the serological results obtained, we used a real-time PCR approach to detect the infection status of the sheep (Groups 1 and 2) by a non-serological method. This is a highly specific test, but its sensitivity is limited by the small size of the sample that can be tested. *T. gondii* parasites have tropism for certain tissues, however, its distribution within one tissue is random, and parasite density may be low. Therefore, a negative result has to be interpreted carefully because it is possible that the parasite could be present in unexamined parts of tissues or in tissues other than those examined. Using a bioassay in mice, Hartley and Moyle (1974) demonstrated *T. gondii* infection in 87% of brain and 55% of muscle samples from congenitally infected sheep [27], whereas Dubey and Sharma (1980) detected the parasite more frequently in skeletal muscle than in any other tissue in experimentally infected sheep [28]. Esteban-Redondo *et al.* (1999) found that *T. gondii* DNA is detected more frequently in ovine brain and heart than in skeletal muscle [29]. In our study, we performed real-time PCR on both, brain and skeletal muscle tissues and increased the sample size from 25 mg (as suggested by the manufacturer) to 1g in order to enhance the chance of detection. However, only five out of six (83.3%) of experimentally infected sheep and 11 out of 18 (61.11%) of naturally infected animals, which were positive by all three serological tests (ELISA, IFAT and IHA), could be confirmed positive by real-time PCR. Four experimentally infected sheep showed positive results in both brain and muscle tissues and one animal only in muscle. From the naturally exposed seropositive animals, a total of four of 18 (22.2%) were confirmed positive only in brain, five of 18 (27.8%) were positive only in muscle and two animals (11.1%) were positive in both examined tissues. According to these results, sampling of more than one type of tissue seems to enhance the chance of *T. gondii* DNA detection.

Performing ROC analysis of results obtained by ELISA on serum and real-time PCR in Group 2, we obtained a relative sensitivity for the ELISA of 100% (at cut-off PP = 20). This is because, in this case, PCR was used as the reference test and all samples that tested positive in PCR were positive in ELISA, and all samples that tested negative in PCR were also negative in ELISA. In this type of evaluation, the relative specificity of the ELISA was comparatively low (91.76%), because some animals that were negative in the PCR were positive in ELISA. These animals could not be confirmed as positive by PCR; however, it is probable that these negative results could be related to the limitations of the PCR method (as discussed above) and positive ELISA results are true.

## Conclusions

At present, sheep are not tested for *T. gondii* infection at slaughter; during meat inspection it is not possible to detect tissue cysts macroscopically and serological tests are not used routinely. Nevertheless, the association between consumption of undercooked lamb or mutton and human infection has been well documented [1,2,7], and different proposals to produce and ensure *T. gondii*-free meat have been discussed [30]. Therefore, improving the surveillance and reporting system for *T. gondii* in sheep populations is important from the public health point of view. The commercial ELISA kit evaluated in this study could represent a valuable tool to collect pre-harvest information at the farm level or, alternatively, for diagnosis at the slaughterhouse, helping to control this widespread zoonosis.

## Competing interests

The authors declare that they have no competing interests.

## Authors’ contributions

SG was involved in the sampling, performing of immunoassays, real-time PCR analysis and statistical evaluation, and drafted the manuscript. RE was involved in the sampling, collection of data and performing of immunoassays. FG contributed to the molecular studies and to the analysis of the results. PD was involved in the design and general supervision of the study, contributed to the analysis of the data and revised critically the manuscript. WB was involved in the design of the study, performing the experimental infections and immunoassays, analysis of the results, drafting and revising the manuscript and supervision of SG. All authors read and approved the final manuscript.

## Supplementary Material

Additional file 1 **Logistic regression between PrioCHECK® Toxoplasma Ab SR ELISA and IFAT performed on serum samples from 96 sheep from Switzerland (Group 2).** PP: ELISA index for percentage of positivity. IFAT values below 1:40 were classified as negative and represented as 1. Click here for file

Additional file 2 **Logistic regression between PrioCHECK® Toxoplasma Ab SR ELISA and IFAT performed on serum samples from 300 sheep from Austria (Groups 3 and 4).** PP: ELISA index for percentage of positivity. IFAT values below 1:40 were classified as negative and represented as 1. Click here for file
