# Supplementary figures and images for: Evaluation of a commercial ELISA kit for detection of antibodies against Toxoplasma gondii in serum, plasma and meat juice from experimentally and naturally infected sheep
Source: Parasit Vectors. 2013 Apr 5;6:85. doi: 10.1186/1756-3305-6-85 (PMC3631134; doi:10.1186/1756-3305-6-85)

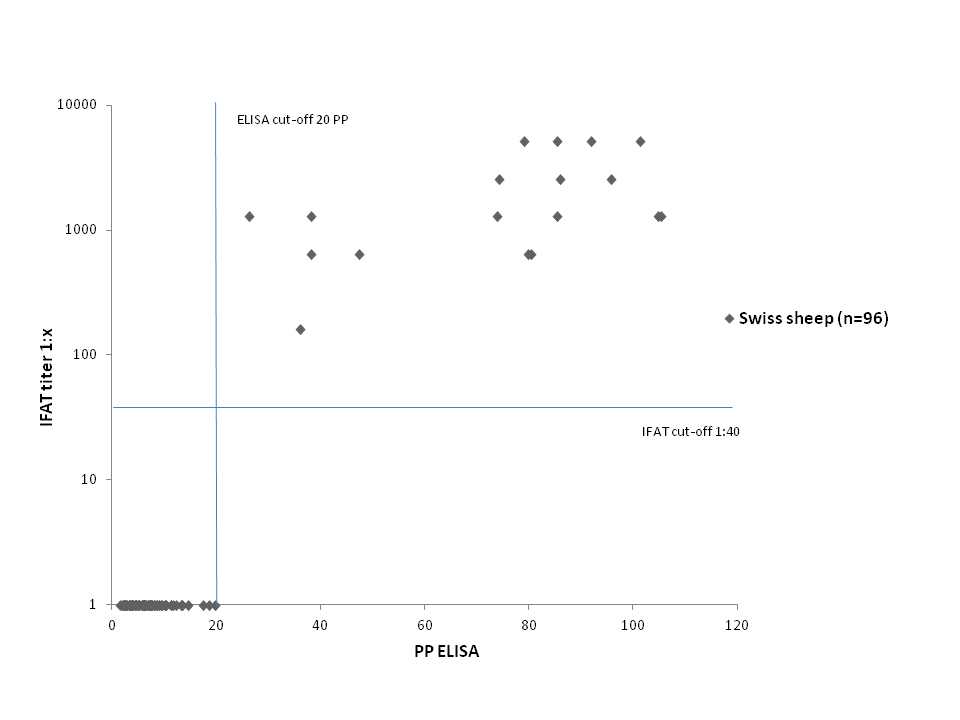

Supplement: Additional file 1 — Logistic regression between PrioCHECK® Toxoplasma Ab SR ELISA and IFAT performed on serum samples from 96 sheep from Switzerland (Group 2). PP: ELISA index for percentage of positivity. IFAT values below 1:40 were classified as negative and represented as 1. [file 1756-3305-6-85-S1.tiff]

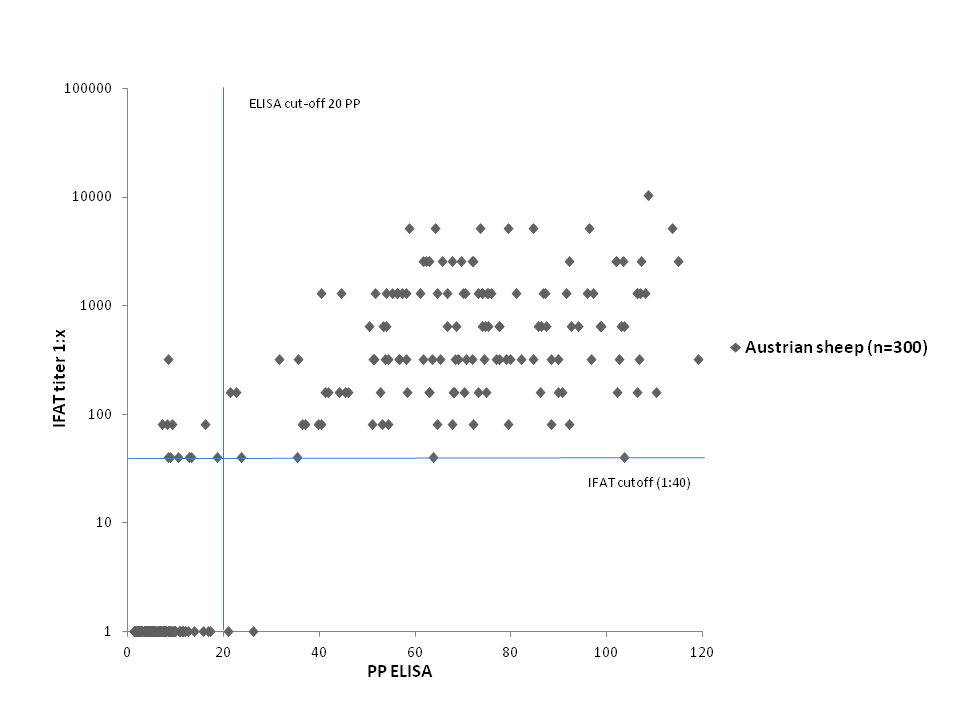

Supplement: Additional file 2 — Logistic regression between PrioCHECK® Toxoplasma Ab SR ELISA and IFAT performed on serum samples from 300 sheep from Austria (Groups 3 and 4). PP: ELISA index for percentage of positivity. IFAT values below 1:40 were classified as negative and represented as 1. [file 1756-3305-6-85-S2.tiff]
